# Exuberant squamous metaplasia of the gastric mucosa in a patient with gastric adenocarcinoma

**DOI:** 10.1186/s13000-015-0281-5

**Published:** 2015-04-30

**Authors:** Sangjeong Ahn, Go Eun Bae, Kyoung-Mee Kim

**Affiliations:** Department of Pathology & Translational Genomics, Samsung Medical Center, Sungkyunkwan University School of Medicine, 81 Irwon-ro, Gangnam-gu, Seoul, 135-710 Korea

**Keywords:** Stomach, Squamous metaplasia, Adenocarcinoma

## Abstract

**Background:**

The presence of squamous epithelium in the stomach is only occasionally encountered and is associated with prolonged mucosal injury. Squamous metaplasia in patients with cancer is relatively rare and only four cases have been reported in the stomach, all of which have been associated with squamous cell carcinomas. We present the first case of exuberant squamous metaplasia in a patient with gastric adenocarcinoma of the cardia.

**Case presentation:**

A 56-year-old woman presented with epigastric pain and weight loss. Endoscopy showed an irregular depressed hyperemic lesion covered with a whitish plaque on the cardia. A total gastrectomy was performed and the tumor in the subcardia was found to extend up to the proximal stomach with diffuse squamous metaplasia in the surface of the tumor and proximal gastric mucosa in contiguity with the esophageal squamous epithelium. It is believed that the squamous extension from the esophagus to the proximal stomach and the gastric adenocarcinoma occurred at the same time.

**Conclusions:**

Synchronous squamous metaplasia and underlying adenocarcinoma in the stomach is extremely rare. Recognition of this entity would be beneficial for clinicians to avoid unnecessary treatment.

**Virtual Slides:**

The virtual slide(s) for this article can be found here: http://www.diagnosticpathology.diagnomx.eu/vs/1035146445160150.

## Background

The presence of squamous epithelium in the stomach is only occasionally encountered and is associated with prolonged mucosal injury [1,2]. However, squamous metaplasia with the coexistence of a neoplasm is relatively rare and only 4 cases have been reported in the English literature—4 cases of squamous cell carcinomas (SCC) and 1 case of SCC in situ [3-7]. However, squamous metaplasia associated with gastric adenocarcinoma has not been reported. We present the first case of exuberant squamous metaplasia in a patient with gastric adenocarcinoma of the cardia.

## Case presentation

**Figure 1:**
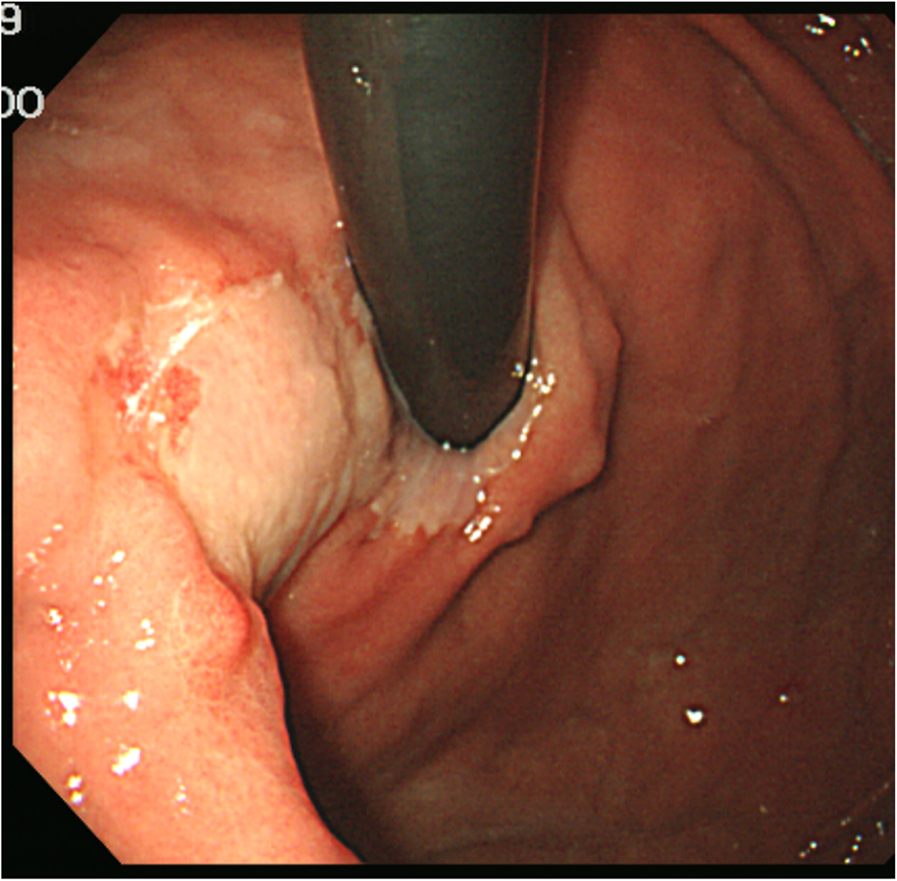
Endoscopic findings of the stomach. An area of white mucosa was found in the cardia contiguous to the esophageal mucosa. On the edge of this area, an irregular and slightly depressed hyperemic lesion was observed.

**Figure 2:**
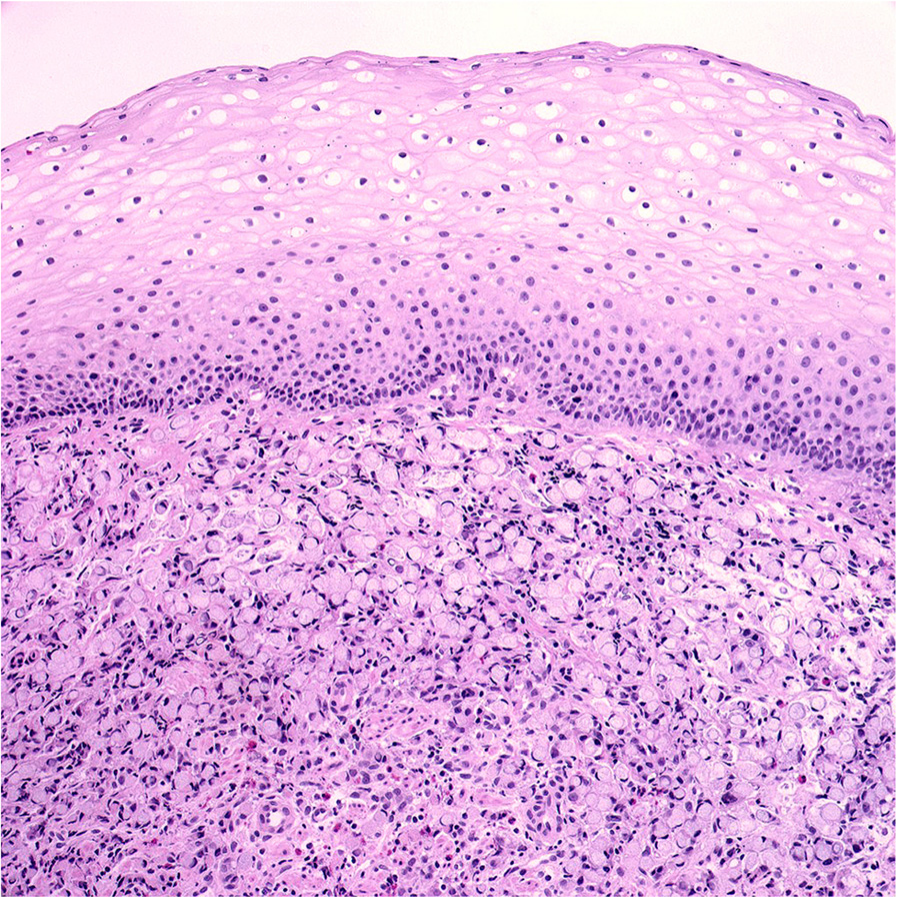
The biopsy specimen showed infiltrating neoplastic signet ring cells beneath benign squamous epithelium.

A 56-year-old Korean woman presenting with epigastric pain and weight loss visited a local clinic and was transferred to our hospital for further treatment. The medical history of the patient did not include any significant prior illness. No significant positive signs were found on physical examination. Endoscopy showed an irregular depressed hyperemic lesion covered with a whitish plaque on the cardia (Figure 1). The lesion was approximately 4 cm in diameter and the whitish plaque was partly contiguous with the esophageal mucosa at the gastroesophageal junction. The biopsy specimen revealed infiltrating neoplastic single cells with benign squamous epithelium (Figure 2). Laboratory results were within normal limits, and serum carcinoembryonic antigen (CEA) levels and carbohydrate antigen 19–9 (CA 19–9) levels were normal. A total gastrectomy was performed without any surgical complications.

**Figure 3:**
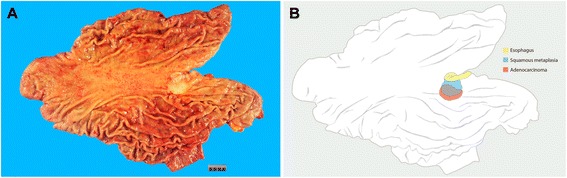
Gross and schematic photos. **(A)** Grossly, an irregular depressed lesion with whitish plaque was detected. (**B)** Histological examination revealed a tumor (red) that was centered in the subcardia extending up to the proximal stomach. Diffuse squamous metaplasia (blue) was present in the surface of the tumor and proximal gastric mucosa in contiguity with the esophagus squamous epithelium (yellow).

**Figure 4:**
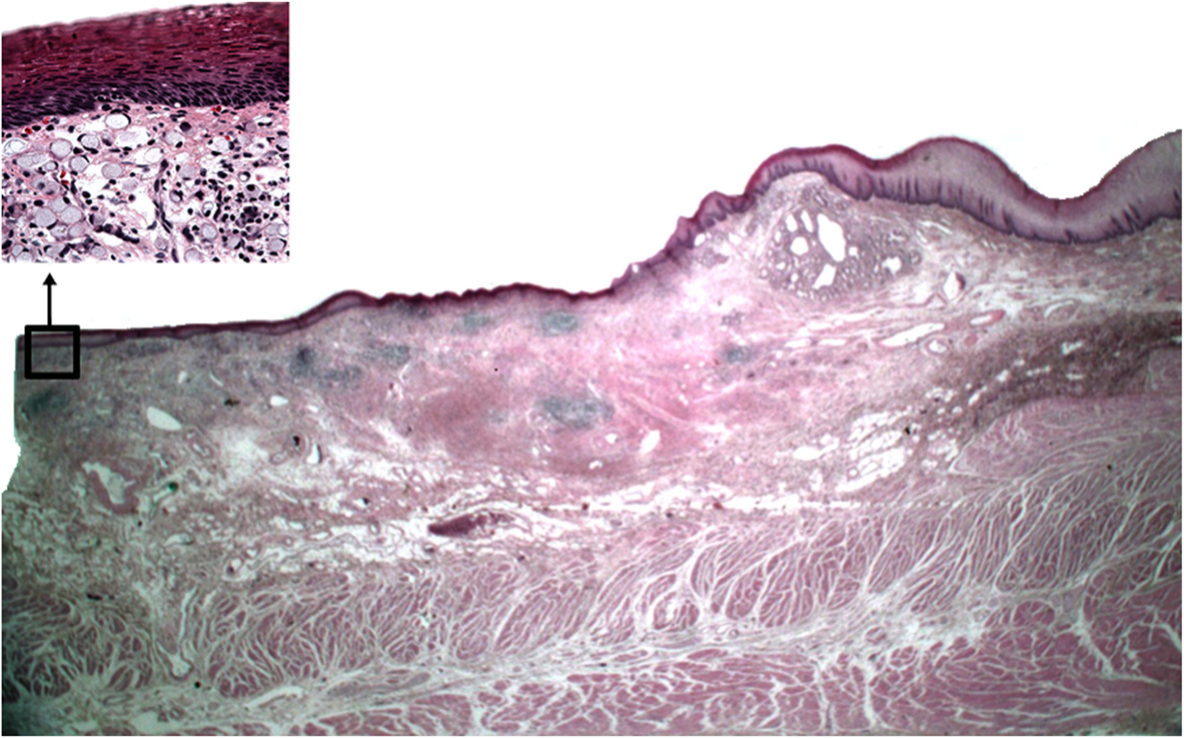
Microscopic findings of total gastrectomy specimen showing esophagus, gastroesophageal junction and squamous metaplasia with underlying gastric adenocarcinoma.

On gross examination, an irregular depressed lesion with a whitish plaque was detected in the high body (Figure 3A). Entire tumor was sampled for histologic examinations. Histological examination revealed poorly differentiated tubular adenocarcinoma that was diffuse-type. The tumor was centered in the subcardia extending up to the proximal stomach without involvement of the gastroesophageal junction (type III by the Siewert classification) [8] (Figure 3B). Diffuse squamous metaplasia was present in the surface of the tumor and the proximal gastric mucosa in contiguity with the esophageal squamous epithelium (Figure 4). We performed EBER *in situ* hybridization and the entire tumor was negative. The tumor infiltrated into the muscularis propria although none of the 54 regional lymph nodes showed metastasis (pT2N0M0, stage IB by the 7^th^ edition of the AJCC/TNM classification). The patient is alive without recurrence 3 month after gastrectomy.

## Discussions

Although the pathophysiology of squamous metaplasia in the stomach remains obscure, prolonged injury appears to be a prerequisite for this mucosal abnormality. The presence of squamous epithelium in the gastric mucosa has been described in patients with underlying diseases such as peptic ulcer [9], tuberculosis [10], syphilis [4], corrosive gastritis [3], pernicious anemia [11], and aberrant pancreatic tissue [12]. These cases support the hypothesized relationship between injurious stimuli and the development of squamous metaplasia. In animal studies, gastric squamous metaplasia has been induced by the injection of pyrogallic acid [13] and methylcholanthere [14]. Squamous metaplasia following chronic irritation occurs elsewhere in the body, for example in the lower respiratory tract, bladder, salivary duct, pancreatic duct, cecum, uterus, and in the mucosa of the prolapsed rectum.

The four patients with squamous metaplasia in the stomach and concurrent carcinoma who have been recorded in the literature are tabulated in Table 1 along with pertinent details given by the authors [3-6]. The stomach from all four patients contained areas of squamous metaplasia transitioning to SCC, corroborating the development of SCC from the squamous metaplasia. The patients in cases 1 to 3 had prior a history of prolonged gastritis provoked by sustained mucosal injuries. The gastric mucosa of the patient in case 1 [3] was damaged by acid and the entirety of the stomach was lined by squamous epithelium. The patient in case 2 [4] was diagnosed with congenital syphilis, progressed to chronic syphilitic gastritis, and had squamous metaplasia in the antrum. The stomach of the patient in case 3 showed diffuse gastritis due to prior cytotoxic chemotherapy for malignant lymphoma [5].

**Table 1:** **Cases of squamous metaplasia with gastric neoplasm reported in the literature**

**No**	**Age/Sex**	**Symptom**	**Location (T/SM)**	**Size (T/SM)**	**Gross findings (T/SM)**	**Associated neoplasm**	**Clinical information**	**Reference**
1	30/M	Dysphagia	Body/body	NA/NA	Ulcerative granular area/NA	Squamous cell carcinoma	History of ingestion of corrosive acid many years earlier	[3]
2	49/F	Epigastric pain	Low body of greater curvature/antrum	NA/2.2 cm	Polypoid mass/whitish irregular mucosal plaque	Squamous cell carcinoma	Congenital syphilis with chronic syphilitic gastritis	[4]
3	55/M	Epigastric pain	Upper body of lesser curvature/anterior wall of cardia	8 cm/NA	Polypoidgray white, granular tumor/patch of grey-white, shiny mucosa	Squamous cell carcinoma	Diffuse gastritis due to prior cytotoxic chemotherapy for lymphoma	[5]
4	71/M	NA	Lesser curvature of Cardia/Lesser curvature of cardia	0.8 cm/NA	Irregular whitish depressed lesion/NA	Squamous cell carcinoma in situ	NA	[6]
5	69/M	Epigastralgia	Lesser curvature of cardia/Lesser curvature of cardia	2.1 cm/NA	Superficial and protruding tumor/whitish mucosa	Squamous cell carcinoma	EBV infection	[7]
Case	56/F	Epigastric pain	Cardia/cardia	4.5 cm/2.5 cm	Irregular depressed lesion/whitish plaque	Adenocarcinoma	Intestinal metaplasia	

T = tumor; SM = squamous metaplasia; NA = not available.

In contrast to these previously reported cases, our case was unique in that the benign squamous epithelium covering the tumor surface was in continuity with the esophageal epithelium without any transition area. Endoscopic and microscopic findings revealed diffuse mucosal atrophy with intestinal metaplasia, which is considered to be a risk factor for gastric adenocarcinoma [15]. Based on the above observations, we hypothesize that the squamous extension from the esophagus to the proximal stomach and the adenocarcinoma occurred at the same time, which is an exceedingly rare event.

The tumor topography of the border between the esophagus and stomach affects the treatment strategy that for patients with type II tumors by the Siewert classification, with esophagectomy offering no advantages over extended gastrectomy [8]. The biopsy specimen in our case showed infiltrating cancer cells beneath the benign squamous epithelium, which could be misinterpreted as adenocarcinoma at the esophagogastric junction or invasion into the esophagus. The endoscopic findings showed gastric extension of whitish plaque in contiguity with the esophagus. Microscopically, the proximal gastric mucosa showed diffuse squamous metaplasia with no evidence of carcinoma. Therefore, the possibility of squamous metaplasia in the stomach should be considered to avoid unnecessary treatment such as proximal esophagectomy. If the squamous metaplasia is too small to recognize, Lugol’s iodine solution would be helpful for staining the metaplastic area [6].

## Conclusions

Synchronous squamous metaplasia and underlying adenocarcinoma in the stomach is extremely rare. Here, we describe the first case with unique endoscopic findings.

## Consent

Written informed consent was obtained from the patient for the publication of this report and any accompanying images.

## Abbreviations

SCC: Squamous cell carcinomas
CEA: Carcinoembryonic antigen
CA 19–9: Carbohydrate antigen 19–9
AJCC: American Joint Committee on Cancer
T: Tumor
SM: Squamous metaplasia
NA: Not available
